# Chemical Observations and Experiments on Air and Fire

**Published:** 1781-05

**Authors:** 


II. *Chemical Obſervations and Experiments on Air and Fire. By* Charles William Scheele, *Member of the Royal Academy at Stockholm, with a prefatory Introduction by* Torbern Bergman. *Tranſlated from the German, by* J. R. Forſter, L. L. D. F. R. S. *and* S. A. *Member of ſeveral learned Societies and Academies in Europe. To which are added Notes by* Richard Kirwan, *Eſq*; F. R. S. *With a Letter to him from* Joſeph Prieſtley, L. L. D. F. R. S. 8vo. London, 260 pages.

MR. Scheele is already known to the world by his ingenious diſcoveries relating to manganeſe, cream of tartar, the acid of arſenic, &c. In the volume before us he has attempted a more lofty, though perhaps a leſs ſucceſsful flight.

He begins his performance with ſome experiments which tend to prove, that beſides aëreal acid, " the air of the atmoſphere is a compound of two kinds of elaſtic fluids." He finds, with other enquirers, that, by phlogiſtic proceſſes, air is diminiſhed. But his explanation of this phenomenon is very different from that of any other philoſopher.

It

It has been imagined by fome that the fame quantity of air is *contracted in bulk* by combination with the phlogifton. By others, that the part of the air loft is *abforbed* by the inflammable fubftance expofed to it. But our author takes it for granted that the air is abfolutely loft, or deftroyed. " I will prove (fays he) that by its " combination with the inflammable principle, " a compound is formed fo fubtle as to pafs " through the fine pores of the glafs, and dif- " perfe all over air." In fine, that fire, or the matter of heat, is the refult of their combination.

Our author difcovered, independent of the ingenious Dr. Prieftley, that dephlogifticated air was obtainable from nitre, and alfo from manganefe, by diftillation. To this air he had given the name of *empyreal air*, by reafon of its property of maintaining combuftion. But in his theory of the origin of this air he differs widely from the philofopher juft now mentioned.

As heat or fire is a compound of this air, and phlogifton, fo he contends that in fome cafes, it is again decompofed, and refolved into its conftituent principles. " The heat employed in the " diftillation of nitre, for example, is compofed of " empyreal air, and the phlogifton of the coals.

" It penetrates the cupel, the ſand, and the re-

" tort, where it meets with the nitrous acid, a

" ſubſtance attracting more powerfully the phlo-

" giſton, than the air does which is united with it;

" conſequently the heat is decompoſed. The ni-

" trous acid acquires a red colour by union with

" its phlogiſton, and the air, which had been di-

" vided into incomprehenſibly minute parts, reaſ-

" ſumes its former quality, and appears under the

" form of empyreal air." The author is ſo far from conſidering this as a mere hypotheſis, that he is convinced by experiments, made in conſequence of it, that it is " one of the cleareſt of truths!"

On this principle he accounts for the reduction of metallic calces by heat. The calx attracts the phlogiſton of the heat, and thereby becomes a metal; the empyreal air remaining in its pure ſtate on the ſurface.—The received, and in our opinion more proper explanations of theſe phenomena are, we conclude, too well known to our readers to need mentioning.

One part of this empyreal air, and three of foul (or, as Doctor Prieſtley terms it, phlogiſticated) air, conſtitute, according to our author, the common air of the atmoſphere. By mixing them in this proportion he found that the properties of the compounds were in all reſpects the

ſame.

fame. Their diminution, on expofure to phlogiftic proceffes, he afcribes to the converfion of the empyreal air (by means of the phlogifton of the inflammatory body) into heat. And his experiments fhew that the quantity of air loft in thefe cafes is exactly equal to that of the empyreal air which the compound contained.

He obferves, that if the procefs proceeds rapidly, the heat generated by the combination of the empyreal air with the phlogifton is very great, as happens in combuftion. Where it goes on more flowly, the heat is lefs apparent, or even infenfible.

The author next proceeds to relate fome curious obfervations which point out a difference in the phenomena of heat that have not hitherto been generally noticed. Thefe obfervations tend to fhew that in combuftion, when fire or heat is generated very rapidly, it paffes off, like light, in right lined directions. It is however different from light: a pane of glafs tranfmits the light, but not the heat. The light may be reflected by a plane mirror; but the heat will remain in the glafs. A polifhed metal plate, however, reflects both the *heat* and the *light*; and (contrary to what happens with glafs) the metal does not grow warm. A focus may be formed with a concave metallic mirror wherein ſulphur may be kindled, and the mirror, by reaſon that it reflects the heat, does not for a long time become warm; whereas if its ſurface be blackened with a candle, "you cannot keep it five minutes "in the ſame ſituation without burning your "fingers."

"If you reflect the heat with a poliſhed "metal plate, to another ſpot, you may, at "the diſtance of ſix feet, form a focus with "it which has ſenſible heat. But if you col- "lect, with a concave mirror, the light re- "flected by a glaſs mirror, the focus will "be very bright, but deſtitute of all heat."

The author gives ſeveral other obſervations on this ſubject, and among the reſt, that this radiant heat (as he very properly terms it) is not leſſened by a current of colder air.

His next inquiry is concerning the nature of *light*. He produces ſeveral experiments, from which he concludes that light contains phlogiſton.—And in ſpeaking of the reduction of *Luna cornua* by light, obſerves, that it is ſooneſt reduced by the violet rays. He contends, that light is nothing more than heat or fire combined with a greater quantity of phlogiſton; and on the ſame principle he accounts for the different colours colours of light. He proceeds further, and aſſerts, that if empyreal air be combined with a ſtill greater quantity of phlogiſton than is neceſſary to conſtitute light, inflammable air is produced.

The author attempts to explain ſeveral chymical phenomena upon theſe principles. But as the theory is evidently ill founded, it will be unneceſſary for us to follow him through this part of his work.

The phenomena exhibited by *aurum fulminans*, is alſo a ſubject of Mr. Scheele's inquiry; and he endeavours to ſhew, that they depend on the volatile alkali. The earth of gold decompoſes this alkali, by depriving it of part of its phlogiſton. The remainder is an air ſimilar to phlogiſticated air; and this, by reſuming its elaſtic ſtate, is, he imagines, the cauſe of the exploſion.

The author now returns to the ſubject of air, and produces experiments from which he concludes, that *empyreal air* is capable of being converted into *aerial acid* or *fixed air*. He conſiders empyreal air as an elaſtic acid, dulcified with phlogiſton. If the phlogiſton be taken from it, it again appears under the form of an acid; and hence he conſiders this air as the the foundation of all acidity. Dr. Prieftley and others are of opinion, that phlogifton is communicated to the air in the lungs. But Mr. Scheele maintains the reverfe of this doctrine. The blood, according to him, attracts phlogifton from the air. The juices of vegetables do the fame in a more powerful manner. The latter, therefore, by taking from the air a greater quantity of the inflammable principle, reduces it to *aerial acid*; whereas blood, attracting that principle lefs powerfully, converts the empyreal air only into phlogifticated air. This kind of air, therefore, he confiders as in a middle ftate between fixed, and empyreal airs; and fuppofes that the three kinds differ from one another only in their containing a greater or lefs quantity of phlogifton.

The fallacy of this doctrine, we imagine, will be fufficiently obvious to our philofophical readers. An experiment which the author brings in confirmation of it (viz. of his having breathed inflammable air a great number of times, and yet wondering that he could not breathe it ftill longer) has been fhewn by the Abbé Fontana to have been founded in error.

Mr. Scheele has difcovered a very ready and ingenious method of detecting the prefence of empyreal empyreal air in water. "I take (ſays he) an "ounce of the water, and let fall into it about "four drops of a ſolution of vitriol of iron, "and add two drops of a ſolution of ſalt of "tartar ſomewhat diluted with water. It im-"mediately yields a dark green precipitate, "which in a couple of minutes changes into "*yellowiſh*, if the water contains empyreal air." —If there be no change from green to yellow, it argues that the water is free from this air. The yellow colour is occaſioned by the phlo-giſton of the precipitate being attracted from it by the empyreal air.

After applying his erroneous theory of heat, &c. to a variety of phenomena, Mr. Scheele concludes his performance with deſcribing a new ſpecies of air which he has diſcovered, and which he calls *ſtinking ſulphureous air*. It is ob-tained by mixing powdered quicklime with an equal quantity of ſulphur, applying a red heat, and catching the air in bladders; and may alſo be procured by other methods. It is with this air, if we remember right, that Mr. Bergman makes the artificial hot mineral water, as the Pyrmont and others are made, with fixed air.

Mr. Kirwan the ingenious annotator on this performance, at the ſame time that he allows his his author all the merit that the new facts contained in his work entitles him to, has with equal candour and ability expofed his miftakes in theory.

Dr. Prieftley in his letter to Mr. Kirwan, at the end of the book, examines the differences of opinions and facts, between himfelf and Mr. Scheele, particularly with regard to the purification of air by vegetables growing in it, the contrary of which is maintained by Mr. Scheele. Thefe points will, we imagine, be more fully difcuffed in Dr. Prieftley's fifth volume of Experiments, which we hear will foon be made public. Profeffor Bergman's preface is chiefly on the beft means of profecuting inquiries in chemiftry, and on the advantages derivable from that ufeful art.

