# Errata

**Published:** 1781-06

**Authors:** 


					.p. 317, 1. 13, fur is alfo read are alfo?

				

